# Application of computational algorithms for single-cell RNA-seq and ATAC-seq in neurodegenerative diseases

**DOI:** 10.1093/bfgp/elae044

**Published:** 2024-11-05

**Authors:** Hwisoo Choi, Hyeonkyu Kim, Hoebin Chung, Dong-Sung Lee, Junil Kim

**Affiliations:** Department of Bioinformatics, Soongsil University, 369 Sangdo-Ro, Dongjak-Gu, Seoul 06978, Republic of Korea; Department of Bioinformatics, Soongsil University, 369 Sangdo-Ro, Dongjak-Gu, Seoul 06978, Republic of Korea; Department of Bioinformatics, Soongsil University, 369 Sangdo-Ro, Dongjak-Gu, Seoul 06978, Republic of Korea; Department of Biomedical Sciences, Seoul National University Graduate School, 103 Daehak-ro, Jongno-gu, Seoul 03080, Republic of Korea; Genomic Medicine Institute, Medical Research Center, Seoul National University, 103 Daehak-ro, Jongno-gu, Seoul 03080, Republic of Korea; Department of Bioinformatics, Soongsil University, 369 Sangdo-Ro, Dongjak-Gu, Seoul 06978, Republic of Korea

**Keywords:** multi-omics, single cell RNA sequencing, single cell ATAC sequencing, data integration, neurodegenerative diseases, multi-modality

## Abstract

Recent advancements in single-cell technologies, including single-cell RNA sequencing (scRNA-seq) and Assay for Transposase-Accessible Chromatin using sequencing (scATAC-seq), have greatly improved our insight into the epigenomic landscapes across various biological contexts and diseases. This paper reviews key computational tools and machine learning approaches that integrate scRNA-seq and scATAC-seq data to facilitate the alignment of transcriptomic data with chromatin accessibility profiles. Applying these integrated single-cell technologies in neurodegenerative diseases, such as Alzheimer’s disease and Parkinson's disease, reveals how changes in chromatin accessibility and gene expression can illuminate pathogenic mechanisms and identify potential therapeutic targets. Despite facing challenges like data sparsity and computational demands, ongoing enhancements in scATAC-seq and scRNA-seq technologies, along with better analytical methods, continue to expand their applications. These advancements promise to revolutionize our approach to medical research and clinical diagnostics, offering a comprehensive view of cellular function and disease pathology.

## Introduction

Neurodegenerative diseases refer to a range of conditions characterized by the progressive degeneration of the structure and function of the nervous system. They are notoriously complex, involving various of genetic, epigenetic, and environmental factors leading to neuronal loss and dysfunction. Alzheimer’s disease (AD) and Parkinson's disease (PD) are two of the most prevalent neurodegenerative disorders. AD is primarily identified by memory loss, cognitive decline, and behavioral changes [1]. Although the exact pathological mechanisms are not fully understood, the accumulation of amyloid-beta has been suggested as a contributing factor. However, various factors likely interact, making the causes and development of AD subjects of ongoing debate. PD is primarily characterized by the loss of dopaminergic neurons in the substantia nigra (SN), a critical area of the brain that controls movement, causing tremors, rigidity, and bradykinesia [2].

A transcriptomic perspective is essential to gain a deeper understanding of these diseases. This approach involves studying the RNA profiles of cells through numerous single-cell RNA sequencing (scRNA-seq) studies. Such studies have provided significant advancements by offering a detailed landscape of gene expression changes in diseased conditions [3, 4]. Single-nucleus RNA sequencing (snRNA-seq), one of the scRNA-seq technology, is particularly beneficial for studying tissues that are challenging to dissociate into individual cells. snRNA-seq excels in analyzing archived or frozen tissues, which are often the only available samples in post-mortem studies [5, 6]. This method enables researchers to utilize valuable archived samples, expanding the potential for major discoveries in neurodegeneration.

**Figure 1:**
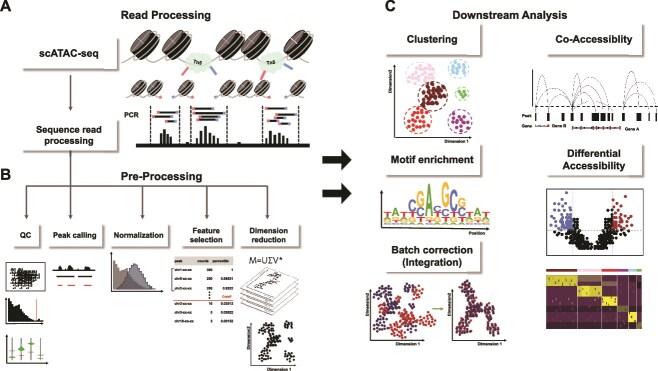
Analysis pipeline of scATAC-seq including preprocessing and downstream analysis.

However, while scRNA-seq illuminates various aspects of gene expression, it has limitations in revealing the complex regulatory mechanisms at the epigenetic level [7]. Epigenetic modifications, such as DNA methylation and histone modifications, play critical roles in regulating gene expression and influencing the progression of neurodegenerative disorders [8]. To complement the insights gained from scRNA-seq and probe epigenetic regulation, researchers have turned to scRNA-seq Assay for Transposase-Accessible Chromatin using sequencing (scATAC-seq) [9, 10]. scATAC-seq is a technique used to assess chromatin accessibility at a single-cell level, revealing insights into the regulatory landscape of individual cells and enabling a detailed understanding of cellular heterogeneity within complex tissues. This technique employs the Tn5 transposase to probe open chromatin regions. The enzyme cuts DNA and integrates sequencing adapters, allowing for subsequent high-throughput analysis. By revealing open and accessible parts of the genome, scATAC-seq helps us understand the regulatory elements driving gene expression changes observed in scRNA-seq studies.

The integration of these two modalities offers a wider view of cellular states and transitions, as it allows researchers to link specific regulatory regions with their corresponding gene expression patterns. This could be crucial in understanding the molecular mechanisms driving cellular responses against various disease states. Neurodegenerative diseases are characterized by complex changes in cellular composition and function within the brain. Integration analysis of scRNA and scATAC sequencing can provide a more comprehensive view of gene regulation at the single-cell level. This deeper understanding of the molecular mechanisms underlying neurodegeneration potentially leads to the discovery of novel therapeutic targets [11–13].

This review paper explores computational methods for analyzing scATAC-seq data and introduces various tools for integrating scATAC-seq with scRNA-seq. By looking into examples where these methods have been put into neurodegenerative diseases, we aim to demonstrate how important they are in helping us get a better grasp of these disorders and how they could lead us toward therapeutic interventions.

## Analysis methods of scATAC-seq

### scATAC-seq data preparation

scATAC-seq utilizes a transposase enzyme to specifically tag open chromatin regions, where DNA is more accessible due to less tight wrapping around histones or other nuclear proteins. (Fig. 1(a)). In the workflow, individual cells are isolated and processed *via* droplet- or plate-based systems [14]. The DNA from the accessible chromatin is then amplified and prepared for high-throughput sequencing, a step that is vital for accurately representing chromatin accessibility data from each cell.

Following the completion of sequencing, the scATAC-seq data undergo an analysis pipeline to transform tens of millions of raw reads into interpretable biological information. Initially, quality control (QC) measures are implemented, including the elimination of reads with poor sequencing quality, adapter sequence trimming, and the application of stringent quality metrics to ensure only the highest-quality reads proceed to the next stages. These curated reads are then aligned to a reference genome using proper tools [15, 16]. A fragment file is generated, showing the coordinates of each chromatin fragment and a barcode to distinguish individual cell.

### Preprocessing of scATAC-seq data

scATAC-seq data, having only 1–10% of open chromatin regions detected in each cell, is even more sparse than scRNA-seq data [7] (Fig. 1(b)). Preprocessing scATAC-seq data demands a thorough and specialized QC strategy. The initial step in QC involves assessing the count depth per barcode to ensure that the cells represented have a balanced and typical number of reads. Barcodes that display either exceedingly low or high read counts are typically indicative of poor-quality cells or potential doublets (multiplets), respectively [17]. Moreover, the Fraction of Reads in Peaks (FRiP) is evaluated, with a higher FRiP indicating effective transposition events [18, 19]. The integrity of the mapping results is further assessed by examining the ratio of reads mapping to promoter regions *versus* blacklist sites—areas known for artifact signals [20]. A higher proportion of reads in promoter regions suggests a higher likelihood of capturing functionally relevant genomic sites. In contrast, an excess of reads in blacklist areas could denote nonspecific transposition or sequencing errors. Another critical quality metric is the enrichment at transcription start sites, where high enrichment signals robust capture of open chromatin regions around key gene regulatory elements [21]. The presence of clear nucleosomal banding patterns, particularly patterns displaying ~200 bp intervals, is another hallmark of quality, indicating well-preserved chromatin structure. Barcodes lacking this distinct pattern are often excluded, as their data may not accurately represent the chromatin accessibility landscape.

Following the QC, scATAC-seq data are arranged as a feature-by-cell count matrix, which is fundamental for all subsequent analyses, as it maps sequencing reads back to genomic locations identified. The matrix arrangement methods can be categorized into peak and bin methods, depending on how regions are delineated. The peak matrix includes rows representing genomic regions with detectable open chromatin peaks across multiple cells, and columns corresponding to individual cells [22, 23]. This approach is particularly valuable for pinpointing high-confidence active regulatory elements. Alternatively, the bin matrix subdivides the genome into fixed-size bins (such as 500 bp or 1 kb) and constructs a matrix where each row represents one of these bins, with columns representing individual cells. This binning method offers a comprehensive view of the chromatin landscape, capturing both highly confident and less accessible regions [7]. After selecting bins or peaks as regions of interest (ROI) in ATAC-seq analysis, the next step involves assigning feature counts. This can be done by counting either the number of fragments overlapping an ROI or the number of insertions within an ROI. Tools like Signac and snapATAC use fragment-based counting [19, 24], while 10X Cell Ranger ATAC and ArchR employ insertion-based counting [14, 25]. Post feature counting, some methods convert the counts into binary 'open' or 'closed' states, though some retain quantitative count data, offering additional insights into nucleosome density (Table 1) [19, 24–44].

**Table 1:** Comparison of various tools for analyzing scATAC-seq.

**Tool**	**Feature Matrix**	**QC**	**Peak calling**	**Dimension Reduction**	**Gene activity**	**Clustering**	**DAR**	**scRNAseq Integration (label transfer)**	**Motif Enrichment (peak)**	**Motif Analysis (score)**	**More Analysis**	**Platform**
**SCRAT [26]**	Peak	O	Conventional bulk peak calling	PCA, t-sne	O	O	O	X	X	X	GUI	R
**chromVAR [27]**	Peak	O	MACS2	t-sne	X	X	X	X	O	O	-	R
**scABC [28]**	Peak	O	MACS2	PCA	O	O	X	X	X	X	Landmarks	R
**BROCKMAN [29]**	K-mer	O	X	k-mer space	O	O	O	X	O	O		R
**Cicero [30]**	Peak	O	MACS2	LSI	O	O	O	X	O	O	co-accessibility	R
**Scasat [31]**	Peak (binary)	O	MACS2	MDS	O	O	O	X	X	X	Batch Correction	Python / R
**Destin [32]**	Peak	O	MACS2	Weighted PCA	O	O	O	X	X	X	-	R
**cisTopic [33]**	Peak	O	MACS2	O	O	O	O	O	O	X	-	Python / R
**scATAC-pro [34]**	Peak/Bin	O	MACS2, GEM, BIN, COMBINED	LSI	O	O	O	O	X	O	GO Analysis, Cicero, Footprinting	Linux / R
**MAESTRO [35]**	Peak	O	MACS2	LSI, PCA	O	O	O	O	O	X	-	R
**ChromSCape [36]**	Peak	O	MACS2	O	O	O	O	X	X	X	GUI, Gene Set Enrichment	R
**SnapATAC [37]**	Bin (5000 bp)	O	MACS2	LDM	O	O	O	O	O	O	Peak-Gene Link	R
**AtacWorks [38]**	Peak (BigWig, BED)	O	Deep Learning Model	PCA	X	O	O	X	X	X	Denoising	Python
**ArchR [39]**	Bin (500 bp)	O	MACS2	Iterative LSI	O	O	O	O	O	O	co-accessibility (Cicero), Footprinting, Pseudo-time (Monocle3), Peak-Gene Link	R
**EpiScanpy [40]**	All (window, Enhancer, promoter, peak,TFBS)	O	X	O	O	O	O	X	X	X	Pseudo-time (PAGA), Differential Methylation Calling	Python
**scOpen [41]**	Peak	O	MACS2	NMF	O	O	O	X	X	X	NMF imputation	R
**Signac [19]**	Peak	O	MACS2	LSI	O	O	O	O	O	O	co-accessibility (Cicero), Footprinting, Pseudo-time (Monocle3), Peak-Gene Link	R
**PeakVI [42]**	Peak	X	X	VAE	X	X	O	X	X	X	Batch Correction Denoising	Python
**epiConv [43]**	Peak	X	MACS2	PCA	O	O	O	O	O	X	Batch Correction	R
**scBasset [44]**	Peak	X	CNN	PCA, LSI	O	X	X	O	X	X	Batch Correction Denoising	Python
**scNCL [45]**	Peak	X	X	t-SNE	O	X	X	O	X	X	-	Python
**snapATAC2 [46]**	Bin (500 bp)	O	MACS3, Bin	spectral embedding	O	O	O	O	O	X	-	Python

For normalizing scATAC-seq data, the TF-IDF (Term Frequency-Inverse Document Frequency) method is highly effective in mitigating biases such as variations in sequencing depth across cells. This approach downweights the influence of peaks commonly observed across many cells and highlights rarer peaks, which provide deeper insights into distinct cellular functions or states. Typically, the term frequency is calculated by dividing the number of counts for a specific peak in a cell by the total counts in that cell, and the inverse document frequency is determined using the logarithm of the ratio of the total number of cells to the number of cells containing that peak. However, the traditional TF-IDF formula often results in a matrix with low variance among nonzero values and a mean close to zero, which is inadequate for differentiating between cell types. To address this limitation, Signac developed a modified TF-IDF formula [19]. In their approach, the IDF is simplified to the ratio of the total number of cells to the number of cells containing the peak. Then, the overall TF-IDF is adjusted by applying a logarithmic scale to a multiplied product of TF and IDF, multiplied by a scaling factor of 10,000.

Feature selection follows normalization. Statistical techniques are employed to select a subset of peaks that either display the greatest variance across cells or contribute most significantly to distinguishing between different cellular phenotypes. Dimensionality reduction is then applied to simplify the complex dataset and extract meaningful patterns. Singular value decomposition, utilized in the Latent Semantic Indexing (LSI) approach, is particularly effective in scATAC-seq. It reduces noise and decomposes the count matrix into singular vectors that capture the most significant variations in the data (Table 1).

### Downstream analysis

Following the preprocessing, the subsequent phase involves elucidating various aspects of gene regulation and cellular functions, providing essential insights into complex biological processes (Fig. 1(C)). Clustering algorithms are employed to organize cells into coherent groups based on similarities in their chromatin accessibility patterns. By identifying clusters of cells with similar chromatin accessibility patterns, researchers can infer potential regulatory mechanisms active in different cellular conditions or states, such as during development, disease progression, or in response to treatments. Graph-based methods such as Louvain and Leiden algorithms are widely used, each differing in their approach but unified in their goal to delineate clusters within the data [45]. These methods detect communities by grouping nodes (cells) that have denser internal connections than connections with the rest of the network, thus revealing natural divisions within the data that can correspond to different cellular states or types.

Differential accessibility region (DAR) identification is foundational in scATAC-seq data interpretation, identifying regions where chromatin accessibility significantly differs between conditions or among cell types. Such differences are critical for understanding the functional discrepancies that may underlie phenotypic changes. Statistical tools such as edgeR [46] and DESeq2 [47], which were originally developed for RNA-seq data, are adapted to address the challenges of counting data in scATAC-seq [48]. Additionally, the Wilcoxon rank sum test and logistic regression are also frequently employed in the analysis of scATAC-seq data [19, 34].

Co-accessibility analysis is employed to identify groups of genomic regions that are frequently open or accessible simultaneously, suggesting potential regulatory interactions like enhancer-promoter connections. This analysis is pivotal for inferring 3D chromatin organization from 2D sequence data, as tools like Cicero model the spatial and co-occurrence relationships of open chromatin regions across cells [30]. Such insights are essential for understanding how interactions between distant genomic regions within the nucleus can regulate gene expression.

Transcription factor motif enrichment involves scanning regions of accessible chromatin for overrepresented DNA sequences that match known transcription factor binding motifs [27]. Identifying these motifs allows researchers to predict which regulatory elements are active in different cell types or under various conditions [23]. Transcription factor footprinting extends beyond identifying motif enrichment by examining the specific patterns of DNA protection within accessible regions, which indicate actual transcription factor binding. Advanced techniques such as PIQ (Protein Interaction Quantification) and TOBIAS (Tool for Integrative Analysis of Bisulfite Sequencing) are utilized to analyze these subtle signatures, which reflect the physical protection of DNA segments from enzymatic cleavage by bound transcription factors [19, 49].

**Figure 2:**
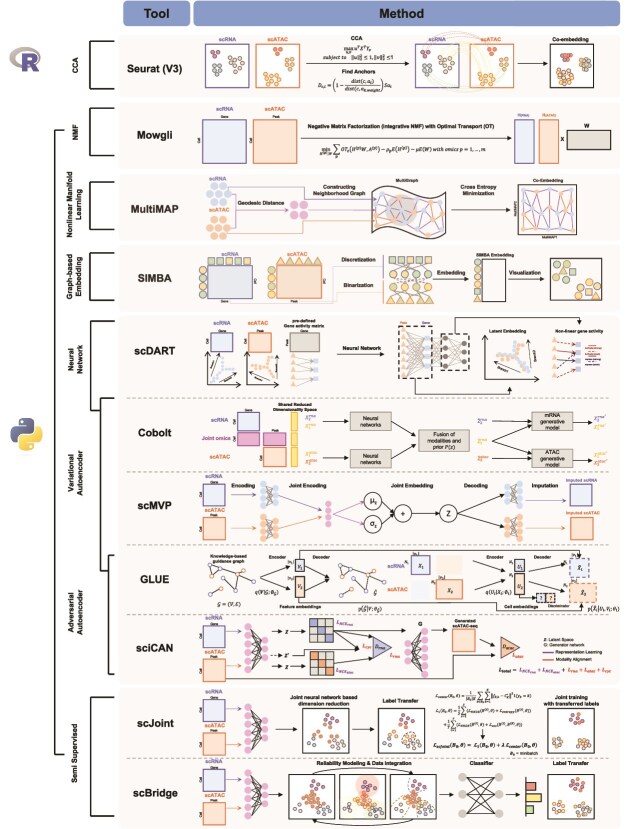
Tools for integrating scRNA-seq and scATAC-seq data.

Collectively, these analyses empower researchers to dissect the intricate layers of gene regulation captured by scATAC-seq.

## Integration of scATACseq with scRNA-seq data

Integrating scATAC-seq with scRNA-seq improves the epigenomic data interpretation by providing a contextual gene expression background. It also helps to refine models of gene regulatory networks, improve cell type identification, and enable more precise predictions of cellular responses to various conditions. This section introduces various computational tools developed for integrating these two modalities (Fig. 2 and Table 2) [50–60].

**Table 2:** Comparison of various tools for integrating scRNA-seq and scATAC-seq data.

**Tool**	**Dataset Type**	**Description**	**Methodology**	**Advantages**	**Limitations**
**Seurat (v3) [53]**	un-paired	- Uses CCA for integrating multi-modal datasets - Utilizes anchors from mutual nearest neighbors for label transfer and integration	CCA	- Well-documented and widely used, making it accessible and easy to implement in various single-cell analyses - Effectively captures the structure of high-dimensional data - Ensures high precision in label transfer onto query datasets	- Weaker performance in aligning paired datasets requiring one-to-one correspondence - Poor performance in batch mixing when integrating data from different datasets
**MultiMAP [54]**	un-paired	- Dimensionality reduction and integration of multimodal datasets through manifold learning - Uses a modified UMAP algorithm to project data by minimizing the cross-entropy on a joint manifold	Nonlinear Manifold Learning	- Excels in label transfer and achieves high clustering performance - Processes data quickly and scales efficiently to handle large datasets - Allows adjusting the influence of each dataset, leveraging unique features in the integration process	- May struggle with complex or noisy data, affecting integration accuracy during nonlinear distortion correction - Can face performance issues with sparse data in graph-based algorithms
**Cobolt [55]**	paired	- Utilizes a multimodal VAE within a hierarchical generative model to jointly model multiple single-cell modalities - Integrates multi-omic single-cell datasets by learning shared latent representations between different data types, facilitating joint analysis - Applicable to both joint-modality datasets and the integration of single-modality datasets with multi-modality datasets	Hierarchical Bayesian Model + VAE	- Improves clustering accuracy by utilizing shared information from multiple modalities - Enhances dimensionality reduction by better capturing complex relationships	- Not scalable for large datasets with many cells - Poor performance compared to unpaired methods when the multiome dataset has a small number of cells
**scMVP [56]**	paired	- Uses a VAE with a GMM prior to create a shared latent representation - Applies self-attention mechanisms to handle sparse chromatin data and mask attention for RNA data	VAE + GMM	- Improves clustering performance by accurately capturing shared features across modalities - Mitigates data sparsity through imputation, enhancing downstream analyses	- Doesn’t generate separate latent space outputs for the two omics - Underperforms in preserving linear trajectories in datasets requiring trajectory conservation
**scJoint [57]**	un-paired	- Utilizes a semi-supervised transfer learning approach, training both labeled and unlabeled data simultaneously through a neural network - Supports label transfer and joint visualization, providing an integrative framework for heterogeneous datasets	Semi-supervised Learning + Neural Networks	- Achieves higher label transfer accuracy in complex atlas-scale datasets - Demonstrates superior clustering performance, leading to more accurate cell groupings	- Relatively weaker performance in omics mixing compared to other methods - Underperforms in preserving linear trajectories in datasets requiring trajectory conservation
**GLUE [58]**	un-paired	- A modular framework integrates unpaired single-cell multi-omics data by using VAEs and a knowledge-based guidance graph - Adversarial learning and iterative optimization help minimize information loss and enhance the alignment of cells across different omics layers	Graph-based VAE + Adversarial Learning	- Consistently superior performance including omics mixing, cell type conservation, and alignment accuracy - Shows strong performance in reducing batch effects, allowing for better integration across different datasets	- Longer running time compared to other methods, especially in smaller datasets
**scDART [59]**	un-paired	- Learns cross-modality relationships using neural networks - Converts chromatin accessibility data into pseudo-RNA-seq matrix before integration	Neural Networks + Gene Activity Function	- Improves gene expression prediction accuracy - Maintains continuous cell trajectories during data integration	- Challenges in integrating datasets with different developmental trajectories across batches - Gene activity prediction from chromatin data is not perfect - Runtime increases significantly with larger datasets
**sciCAN [60]**	un-paired	- Integration using cycle-consistent adversarial network - Does not require anchors for integration	Cycle-Consistent Adversarial Network	- Suitable for non-jointly profiled single-cell data - Preserves biological variation while removing modality differences - Consistent performance in label transfer	- Potential instability in training due to cycle-consistent loss and adversarial learning - Involves high computational complexity
**scBridge [61]**	un-paired	- Merges annotated scRNA-seq and unlabeled scATAC-seq data using a semi-supervised, deep learning-based approach. - Leverages variable correlations between chromatin accessibility and gene expression for integration	Semi-supervised Learning + Heterogeneous Transfer Learning	- Progressively integrates reliable ATAC-seq cells by iterative selection - Improves integration accuracy by selecting the most reliable cells	- Incorrect initial cell selection can impair integration performance - High heterogeneity reduces integration efficiency
**Mowgli [62]**	paired	- Combines integrative NMF and OT - Enhances embedding and clustering quality with high biological interpretability across various platforms	NMF + OT	- Captures similarities across datasets for improved integration and clustering performance	- Increases computation times due to the complexity of combining NMF and OT
**SIMBA [63]**	un-paired	- Joint embedding of cells and genomic features in a single graph - Addresses batch effects and supports marker discovery without clustering	Graph-based Embedding + Softmax Transformation + Weight Decay	- Handles multiple single-cell tasks within a single framework - Effectively identifies relationships between cells and features using a graph-based approach	- Involves complexity with graph-based methods - Computational challenges with large datasets

**Seurat (v3)** [50] starts with pre-processing, which includes normalizing and scaling both the scRNA-seq and scATAC-seq datasets. The method primarily uses canonical correlation analysis (CCA) for integration, which captures the shared feature correlation structure between two modalities. This approach involves finding anchors from mutual nearest neighbors across datasets, which are then used to transfer labels and integrate the datasets into a unified representation. By leveraging CCA and LSI, Seurat can capture the underlying structure of the data, ensuring effective integration and accurate downstream analysis.

**MultiMAP** [51] is a robust integration tool designed for dimensionality reduction and integration of multimodal datasets. It operates by projecting different datasets into a shared low-dimensional space, preserving the manifold structure of the data. It normalizes intra-dataset distances based on specific neighborhood distances and inter-dataset distances in a shared feature space. These distances construct a neighborhood graph, which is projected into a low-dimensional embedding space by minimizing cross entropy.

**Cobolt** [52] uses a hierarchical Bayesian generative model combined with multimodal variational autoencoder (VAE) to integrate datasets, even when cells do not share the same modalities, making it versatile for diverse biological inquiries.

**scMVP** [53] integrates scRNA-seq and scATAC-seq data by creating a shared latent space using a Gaussian mixture model within a VAE framework. This model inputs raw scRNA-seq counts and TF-IDF transformed scATAC-seq data to derive a common latent embedding, which is used for clustering, imputation, and downstream analysis. The process involves attention-based neural networks to capture correlations within and between the data types. The model is trained with a back-propagation algorithm.

**scJoint** [54] employs neural networks to create joint embeddings of scRNA-seq and scATAC-seq data, capturing both shared and unique features. scJoint employs a semi-supervised approach to co-train labeled scRNA-seq and unlabeled scATAC-seq data by aligning these distinct modalities in a common lower-dimensional space. The process involves three main steps. First, joint dimension reduction and modality alignment are achieved using a neural-network-based dimension reduction loss, which extracts orthogonal features with maximal variability, and a cosine similarity loss, which aligns projections from both modalities. This step also includes a cell-type classification loss to guide the scRNA-seq embedding. In the second step, scATAC-seq cells are treated as queries, and k-nearest neighbors among scRNA-seq cells are identified in the common embedding space, transferring cell-type labels from scRNA-seq to scATAC-seq by majority vote. The third step enhances the mixing of modalities using a metric learning loss with the transferred labels.

**GLUE** [55] is a computational framework designed for integrating single-cell multi-omics data, including scRNA-seq and scATAC-seq, especially when they are un-paired. The framework employs VAEs to generate low-dimensional embeddings for each omics type, which are then linked through a guidance graph based on known regulatory interactions. GLUE aligns these embeddings using adversarial learning, ensuring that the representations of different omics data are consistent.

**scDART** [56] is an integration tool designed to merge unmatched scRNA-seq and scATAC-seq data. Its primary method involves learning cross-modality relationships using neural networks to better represent and integrate these disparate data types. It includes a gene activity function module that converts chromatin accessibility data into a pseudo-scRNA-seq matrix and a projection module that integrates this with actual scRNA-seq data into a shared latent space. This approach contrasts with traditional methods that use predefined linear transformations or manifold alignment.

**sciCAN** [57] is a novel method that integrates chromatin accessibility and gene expression data using a cycle-consistent adversarial network. Unlike previous methods, sciCAN does not require cell anchors, making it suitable for non-jointly profiled single-cell data. It comprises two main components: representation learning and modality alignment. The encoder extracts feature into a joint low-dimensional space using noise contrastive estimation, while two discriminator networks facilitate modality alignment through adversarial domain adaptation and cycle-consistent loss.

**scBridge** [58] is a semi-supervised integration tool that merges annotated scRNA-seq data with unlabeled scATAC-seq data using a heterogeneous transfer learning approach. This method exploits the variable correlations between chromatin accessibility in scATAC-seq cells and gene expression in scRNA-seq cells to facilitate integration. The process starts by training a deep neural encoder and classifier using annotated scRNA-seq data, followed by estimating the reliability of scATAC-seq cells using a Gaussian mixture model (GMM) based on discriminability and classification confidence. Reliable scATAC-seq cells are then integrated with scRNA-seq cells through cross-omics prototype alignment. By iteratively selecting and merging the most reliable scATAC-seq cells, scBridge progressively narrows the modality gap, leading to a refined and integrated dataset​.

**Mowgli** [59] is an integrative method for analyzing single-cell multi-omics data, combining integrative Non-negative Matrix Factorization (NMF) with Optimal Transport (OT). This approach leverages the intuitive part-based representation of NMF, enhancing interpretability, while OT improves clustering performance by capturing similarities between single-cell omics profiles. Mowgli has been extensively benchmarked against state-of-the-art methods, demonstrating superior embedding and clustering quality, particularly in terms of biological interpretability, when applied to data from platforms like CITE-seq [61], 10X Genomics Multiome, and TEA-seq [62]. This method can handle paired multi-omics datasets of any type and number without specific statistical assumptions.

**SIMBA** [60] embeds cells and their genomic features into a shared latent space, enabling comprehensive single-cell analyses. Unlike existing methods focusing only on cell states, SIMBA treats cells and features as nodes in a single graph. Essential procedures in SIMBA include Softmax transformation for data normalization, weight decay to prevent overfitting, and entity-type constraints to ensure accurate co-embeddings of cells and features. SIMBA constructs a graph where nodes represent various entities and edges represent experimentally measured or computationally inferred relationships. For example, an edge between a gene and a cell signifies gene expression in that cell, with the edge weight reflecting expression levels. Computationally inferred edges model regulatory interactions. SIMBA processes this graph to create a low-dimensional representation of nodes, capturing cellular heterogeneity and enabling the discovery of cell-type-specific features without clustering. The proximity of nodes in this space indicates the likelihood of relationships, aiding in identifying marker genes and regulatory elements​.

Comparative studies of tools for integrating scRNA-seq and scATAC-seq data, including some of the methods discussed in this review, highlight the distinct advantages each tool offers [12, 13]. For un-paired datasets, GLUE was the leading performer, while Seurat demonstrated strong results in mixing omics data within a 2D space. Meanwhile, scJoint and scDART excelled in preserving cell type identity. For paired datasets, most methods performed well, especially with smaller cell numbers. However, as datasets grew larger in terms of both cell numbers and cell type diversity, some methods struggled to maintain their effectiveness. Notably, GLUE outperformed others in combining different types of omics data types, preserving cell types, and aligning the data accurately. Regarding trajectory conservation, scMVP and scDART scored well on specific metrics, underscoring their utility in this aspect. When selecting an integration method, it is important to consider the dataset's category and size comprehensively. For instance, Seurat and scDART are recommended for omics mixing, while scMVP is a strong choice for cell-type conservation. Additionally, Seurat, scJoint, and scDART offer scalable solutions with user-friendly interfaces, further enhancing their applicability.

## Applications of scRNA and scATAC-seq in neurodegenerative disease

### Applications in AD

The advanced computational tools for scRNA and scATAC-seq allow to elucidate how chromatin conditions and gene expression affect the disease. Especially in neurodegenerative disease, recent research employing scATAC-seq has provided profound insights into the molecular changes associated with AD, significantly advancing our understanding of its complex pathology (Table 3).

**Table 3:** Application of single-cell multi-omics in neurodegenerative study.

**Disease**	**Single cell Data**	**Method**	**key findings**	**Reference**
**AD**	Prefrontal cortex, 130,418 nuclei: snATAC-seq (12 late-state AD, 8 control) 61,472 nuclei: snRNA-seq (11 late-state AD, 7 control)	UMAP, Leiden clustering, TF motif analysis, DEGs, RNA-ATAC integration, GO analysis, co-accessibility analysis, regulatory network	APOE, CLU, SREBF1, and associated cREs	[63]
	snATAC-seq (12 AD and 8 control) snRNA-seq (11 AD, 7control)	Seurat workflow, Signac workflow, UMAP, Leiden clustering, TF motif analysis, DEGs, co-accessibility analysis	CDK18, FOXP1, ANKRD44, KAZN, and associated cREs	[64]
	snATAC-seq, snRNA-seq (12 late-onset Alzheimer’s disease (LOAD), 12 control)	Seurat workflow, UMAP, Signac workflow, Multiplet detection, RNA-ATAC integration, Peak calling, DEGs, DAPs, co-accessibility analysis, GO analysis, TF motif analysis	APOE, MYO1E, BIN1, ELK1, JUN, SMAD4, and associated cREs	[65]
	Dorsolateral prefrontal cortex (DLPFC), 105,332 nuclei: snATAC-seq, snRNA-seq (7 AD, 8 control)	Seurat workflow, Signac workflow, RNA-ATAC integration, WNN analysis, DEGs, Gene set enrichment, Peak calling	ZEB1, MAFB, and associated cREs	[66]
**PD**	SN, 55,937 nuclei: snATAC-seq (8 late-stage PD, 7 control), 57,270 nuclei: snRNA-seq (13 late-stage PD, 6 control)	Seurat workflow, Signac workflow, Multiplet detection, UMAP, Louvain clustering, DEGs, DARs, GO analysis,	SNCA, LRRK2, MAPT, AGTR, and associated cREs	[67]
	SN, 69,289 nuclei: snATAC-seq, snRNA-seq (14 PD, 9 young-control, 8 aged-control)	Seurat workflow, Signac workflow, Multiplet detection, UMAP, Louvain clustering, DEGs, DARs, Pseudo-time analysis, Motif enrichment analysis	CARNS1, MAPT, and associated cREs	[68]
	SN, 481,703 nuclei: snATAC-seq, snRNA-seq (19 PD, 14 control)	Seurat workflow, UMAP, DEGs, Louvain clustering, ArchR workflow, RNA-ATAC integration, gene-TF regulatory network	CD83, HIF1A, and associated cREs	[69]
**AD, PD**	Isocortex, Striatum, Hippocampus, SN, 70,631 cells: scATAC-seq (10 Healthy brain)	Seurat workflow, UMAP, Louvain clustering, Gene activity scores, DARs, TF motif analysis	BIN1, MAPT, MAPT, and associated cREs	[70]

**Morabito *et al.*** [63] utilized advanced methodologies to investigate the gene-regulatory landscape of the brain in AD. The researchers conducted a multi-omic single-nucleus analysis on 191,890 nuclei from the prefrontal cortex of late-stage AD patients, profiling chromatin accessibility and gene expression concurrently. This approach revealed significant cellular heterogeneity and identified cell-type-specific candidate cis-Regulatory Elements (cREs) and target genes, including *APOE* and *CLU* linked to oligodendrocytes. They constructed co-accessibility maps to reveal regulatory relationships at AD risk loci based on genome-wide association studies (GWAS). The study also used trajectory analysis to uncover disease-relevant transcription factors like *SREBF1* in glial populations and employed single-nucleus consensus-weighted gene co-expression analysis for network analysis despite sparse single-cell data. This comprehensive study provided critical insights into the cell-type-specific regulatory mechanisms in AD, highlighting genes such as *SREBF1*, *APOE*, *CLU*, and others, thereby offering potential therapeutic targets​.

**Xu *et al.*** [64] conducted a comprehensive analysis to map the regulatory network anomalies associated with AD. They used the single-nucleus sequencing data introduced earlier and employed techniques such as scATAC-seq to profile chromatin accessibility and ChIP-seq to identify active enhancers. These datasets were integrated with scRNA-seq data to map out the transcriptional regulatory networks involved in AD. They annotated enhancers and other regulatory elements, which were linked to gene expression changes observed in AD. By constructing 3D chromatin interaction maps using Hi-C, they identified significant enhancer-promoter interactions and pinpointed regulatory elements associated with key AD genes. The study identified several key genes, including *CDK18*, *FOXP1*, *ANKRD44,* and others, that are regulated by these enhancers.

**Gamache *et al.*** [65] utilized advanced snRNA-seq and scATAC-seq on nuclei from 12 normal and 12 AD brains. This study identified cell subtype-specific clusters, DEGs, DARs, and cis co-accessibility networks. Integrative analysis revealed disease-relevant regulatory networks and candidate genes, such as *APOE*, *MYO1E*, and *BIN1*, highlighting dysregulations in excitatory neurons and microglia. The findings underscored the role of specific transcription factors (*e.g. ELK1*, *JUN*, *SMAD4*) in AD pathogenesis.

**Anderson *et al.*** [66] utilized 10x Genomics Multiome technology to analyze gene expression and chromatin accessibility in the dorsolateral prefrontal cortex of 7 AD patients and 8 control donors. The sequencing data were then integrated and analyzed using Seurat for transcriptomic data and Signac for chromatin accessibility data. Through these methods, the researchers identified 36 distinct cell clusters, which included various neuronal and non-neuronal cell types. By integrating gene expression and chromatin accessibility profiles, they discovered key regulatory elements and transcription factors associated with AD. Notably, they identified *ZEB1* and *MAFB* as critical regulators of AD-specific cREs.

### Application in PD

Here, we introduce recent studies employing scATAC-seq provide deep insights into the molecular mechanisms altered in PD. The highlighted articles specifically focus on characterizing the molecular changes and identifying new targets for potential therapeutic intervention (Table 3).

**Lee *et al.*** [67] conducted a comprehensive analysis using various advanced methodologies and tools to investigate the molecular underpinnings of PD at a cellular level. The researchers collected brain samples from the SN of both healthy controls and PD patients and employed snRNA-seq and scATAC-seq to profile 113,207 nuclei. By integrating the multi-omics data, the study annotated 128,724 cREs specific to different cell types within the SN. High-resolution 3D chromatin contact maps were constructed using *in situ* Hi-C, identifying 656 target genes of dysregulated cREs and genetic risk loci associated with PD. Distinct modular gene expression patterns across different cell types, particularly highlight alterations in dopaminergic neurons and glial cells such as oligodendrocytes and microglia. Notably, the research identified several key genes implicated in PD, including *SNCA*, *LRRK2*, *MAPT*, and *AGTR*.

**Adams *et al.*** [68] employed snRNA-seq and scATAC-seq to profile midbrain samples from young, aged, and PD patients. The researchers analyzed shared gene expression and chromatin accessibility to identify age- and disease-related changes. The study highlighted significant alterations in glial cells, particularly microglia and oligodendrocytes. Notably, a novel disease-associated oligodendrocyte subtype was discovered, along with key genes such as *CARNS1* and *MAPT*. Peak-gene association analysis identified 89 PD-associated SNP loci, with five located in *MAPT*, showing differential expression in disease-associated oligodendrocytes. These findings underscore the pivotal role of glial cells in PD pathogenesis and aging.

**Chatila *et al.*** [69] employed a multi-omics approach to investigate microglial heterogeneity in PD. The researchers utilized snRNA-seq and scATAC-seq on postmortem brain samples from 19 PD donors and 14 non-PD controls. This analysis was conducted using the 10x Genomics platform to create transcriptomic and epigenomic profiles of the SN and other brain regions affected by PD, including the ventral tegmental area, substantia innominata, and hypothalamus. The study identified thirteen distinct microglial subpopulations, along with perivascular macrophages and monocytes, and characterized their transcriptional and chromatin landscapes. Through this comprehensive profiling, the researchers discovered several microglial subpopulations associated with PD, noting significant regional specificity. A notable finding was the depletion of a specific microglial subpopulation expressing *CD83* and *HIF1A*, which was particularly prevalent in the SN of PD patients. This subpopulation showed a unique chromatin signature and was enriched for transcripts involved in antigen presentation and heat-shock proteins. The depletion of this CD83+ microglial subpopulation in the SN suggests a potential role in the increased neuronal vulnerability observed in PD. This study highlights the importance of microglial diversity and regional specificity in the pathogenesis of PD, providing new insights into the neuroinflammatory processes that may drive neurodegeneration.

### Application in both AD and PD

**Corces *et al.*** [70] present a high-resolution epigenetic characterization of inherited noncoding variations associated with AD and PD by employing an integrative multi-omics framework with a machine-learning classifier. To gain insights into brain-region and cell-type-specific chromatin accessibility, scATAC-seq was performed on 10 brain samples, encompassing the isocortex, striatum, hippocampus, and substantia nigra. This approach successfully predicted dozens of functional SNPs, identifying gene and cellular targets for each noncoding GWAS locus. It highlights well-known disease-associated genes such as *BIN1* in AD while suggesting novel gene-disease associations like *STAB1* in PD. Moreover, the study dissected the complex inverted haplotype of the *MAPT* (encoding tau) Parkinson’s disease risk locus, identifying putative ectopic regulatory interactions in neurons that may mediate the disease association. This comprehensive approach implicates approximately five times as many genes in the genetic association with AD and PD, expanding our understanding of noncoding contributions to these neurodegenerative diseases.

### Summary of the applications in neurodegenerative disease

The application of scATAC-seq has significantly advanced our understanding of the molecular mechanisms underlying AD and PD in terms of cell type-specific regulatory elements and potential therapeutic targets. The cell type-specific molecular mechanism cannot be identified by the bulk sequencing approach. For AD, research has revealed significant cellular heterogeneity in the brain, identifying key genes such as *APOE*, *CLU*, *SREBF1*, and transcription factors like *ZEB1* and *MAFB* that are implicated in disease pathogenesis, particularly in oligodendrocytes and glial cells. For PD, key genes such as *SNCA*, *LRRK2*, *MAPT*, and the discovery of disease-associated oligodendrocyte subtypes and specific microglial subpopulations suggest a pivotal role of glial cell dysregulation and neuroinflammation in PD.

## Challenges and future directions

The application of scRNA and scATAC-seq in neurodegenerative disease research, while transformative, is not without its challenges. One of the primary hurdles is the technical complexity involved in obtaining high-quality, single-cell chromatin accessibility data. This is particularly challenging in the context of neurodegenerative diseases, where sample availability and quality can be limiting factors. Additionally, the sheer volume of data generated by scATAC-seq experiments necessitates advanced computational tools for effective analysis and interpretation. Current tools and methods may require further refinement to fully exploit the depth of the data, manage batch effects, and integrate with diverse datasets. Integrating scATAC-seq with scRNA-seq also presents several significant challenges. One major challenge is batch effects, arising from differences in experimental conditions, sequencing platforms, or sample processing, which further complicate integration by introducing variability that can obscure true biological signals. Managing these effects is critical for accurate data integration, especially when datasets are generated from different laboratories or time points. Moreover, aligning datasets from these two omics layers requires sophisticated computational methods capable of bridging the gap between gene expression profiles and chromatin accessibility landscapes. This involves the development and refinement of algorithms that can link open chromatin regions identified by scATAC-seq with the corresponding gene expression patterns captured by scRNA-seq.

Looking forward, several promising directions could significantly enhance the impact of scATAC-seq in neurodegenerative disease research. First, the integration of scATAC-seq data with other single-cell omics approaches, such as scRNA-seq and single-cell proteomics, could provide a more comprehensive view of the cellular changes occurring in neurodegenerative diseases. Such integrative approaches would help in elucidating the complex regulatory networks at play and pinpointing potential therapeutic targets. However, the batch effects and technical variability between experiments can exacerbate these integration challenges, making it difficult to align datasets accurately. In contrast, multi-omics technologies like DOGMA-seq [71] and TEA-seq [62], which simultaneously capture transcriptomic, epigenomic, and proteomic data from the same single cells, offer a significant advantage. These single-cell multi-omics technologies can serve as an anchor dataset linking the fragmented information obtained from single-omics studies of RNA, chromatin, and protein [72]. This comprehensive and unified dataset allows for a more precise identification of complex regulatory mechanisms.

Furthermore, advances in artificial intelligence (AI) have the potential to improve data analysis by enabling more precise modeling of disease progression and therapeutic responses. Recently developed tools such as scGPT [73] and scBERT [74] utilize large language models (LLMs)—AI models pre-trained on vast datasets—to integrate diverse data sources and learn foundational representations of cellular states and processes. This approach improves prediction accuracy and facilitates the discovery of new biological insights by integrating previously siloed data sources. Recent advancements highlighted in the LLMs include its ability to predict cellular responses and integrate multi-modal datasets. For instance, the model can predict how specific genetic perturbations will alter cellular behavior, thus helping the development of personalized medicine strategies. These advances illustrate how AI is not only enhancing the precision of data analysis in biomedical research but also enabling the integration of complex and diverse data types, leading to more comprehensive models of disease progression and therapeutic responses.

Moreover, as technology advances, increasing the throughput and resolution of scATAC-seq will allow for broader studies with larger sample sizes. This scalability is crucial for capturing the full heterogeneity of disease states across diverse patient populations. Translating these research insights into clinical settings could pave the way for precision medicine applications, offering tailored therapeutic strategies based on individual epigenomic profiles.

## Conclusion

scATAC-seq has profoundly reshaped our understanding of the epigenetic dynamics in neurodegenerative diseases. As demonstrated throughout this review, this technology offers an unprecedented window into the chromatin accessibility landscape at the single-cell level, revealing the intricacies of gene regulation that underlie the pathogenesis of conditions such as AD and PD. By integrating scATAC-seq with scRNA-seq data, researchers can gain a more comprehensive view of gene expression and regulation, enhancing our understanding of these complex diseases. Specifically, several studies in AD and PD using scATAC-seq and scRNA-seq have discovered candidate cREs and genes, including *APOE*, *CLU*, and *SNCA*, as well as transcription factors such as *ZEB1* and *MAFB*. The insights gained through these combined approaches have not only deepened our understanding of neurodegenerative diseases but have also unveiled novel biomarkers and therapeutic targets. Adding other single-cell omics, such as proteomics and advances in scATAC-seq analysis using AI, may lead to a better understanding of the diverse intracellular environments that play a regulatory role, which could help in the clinical process for therapeutics. These advancements suggest a promising horizon for personalized medicine, with the potential to significantly impact future research and clinical applications.

**Key Points**

- The analysis pipelines for scATAC sequencing data analysis were explored, covering aspects such as data preparation, pre-processing, and downstream analysis.
- Integrating scATAC sequencing with scRNA sequencing data enables elucidation of gene regulatory mechanisms.
- The application of single-cell multi-omics on neurodegenerative diseases facilitates in uncovering novel molecular mechanisms for disease progression and their clinical applications.

## Author Biographies

**Hwisoo Choi** He is an undergraduate student at the Department of Bioinformatics, Soongsil University. His research interests focus on tool development for integrating scRNA-seq and scATAC-seq and its application in Parkinson’s disease.

**Hyeonkyu Kim** He is a PhD Candidate at the Department of Bioinformatics, Soongsil University. His research interests include tool development for integrating scRNA-seq and scATAC-seq and atlas-scale cardiovascular single cell analysis.

**Hoebin Chung** He is a Master Candidate at the Department of Bioinformatics, Soongsil University. His research interests focus on tool development for integrating scRNA-seq and scATAC-seq and its application in thyroid cancer.

**Dong-Sung Lee, PhD** Dong-Sung Lee is an Associate Professor at the University of Seoul, specializing in genomics and epigenomics. He has developed innovative single-cell sequencing techniques and has extensive experience in bioinformatics and molecular biology.

**Junil Kim, PhD** He is an Assistant Professor at the Department of Bioinformatics, Soongsil University. His research interests focus on tool development for integrating single cell multi-omics and spatial transcriptomics data.
